# Affections of Sight from Injuries1Read, May 12, 1891, before the Fourth Branch of the New York State Medical Association.

**Published:** 1891-10

**Authors:** Alvin A. Hubbell

**Affiliations:** Professor of Ophthalmology and Otology, in the Medical Department of Niagara University, Buffalo, N. Y.; 212 Franklin Street


Buffalo Medical and Surgical Journal

VOL. XXXI. OCTOBER, 1891. No. 3.

### Original Communications.

### AFFECTIONS OF SIGHT FROM INJURIES.[1]

BY ALVIN A. HUBBELL, M. D.,

Professor of Ophthalmology and Otology, in the Medical Department of Niagara University, Buffalo, N. Y.

THE results of injuries in general are very various, and often most complex and obscure. It requires a broad acquaintance and great familiarity with the multiformity of contusions, punctures, lacerations, fractures, incisions, and concussions which may be inflicted upon the different parts and structures of the human body, in order to truly estimate their effects, and to direct and carry out the proper treatment. Permit me, therefore, to introduce for discussion at this time a subject that is not new, but that will profitably bear reconsideration, especially in the light of certain researches that have recently been made in the anatomy, physiology, and pathology of the central nervous system, viz., that class of injuries that may affect vision.[2]

Those injuries which may affect vision may do so directly or indirectly; immediately or after the expiration of a period of time; temporarily or permanently.

*Directly*, an injury usually affects vision by implicating the eyeball or its muscles. It may, however, involve the optic nerve, and even the visual path within the cranium, viz., the optic tract and the optic fibers within the basal ganglia, internal capsule, and "radiations of Gratiolet" in the white substance of the brain, and, also, the visual center in the cortex of the occipital lobe. Concussions, contusions, and hemorrhages may produce the lesions, or they may arise less remotely by lacerations, cuts, and pressure directly inflicted by the missile, foreign substance, or displaced bone.

---

1. Read, May 12, 1891, before the Fourth Branch of the New York State Medical Association.

2. It will not be in keeping with the brevity at which I aim to introduce in this paper illustrative cases, although I would be glad to do so.

*Indirectly*, the visual apparatus may become affected by pressure through other parts, by reflex influences, by disturbances of the sympathetic nervous system, and by extension from a distant point of injury of some pathological process, such as inflammation or degeneration due to vaso-motor or other changes. Almost any part of the visual apparatus may thus suffer.

*Immediate* impairment of vision may come from both direct and indirect injuries, but it usually follows direct, while that which comes on after the lapse of more or less time, usually follows indirect.

The vision is more likely to be *temporarily* affected in those injuries which disturb it immediately, while its loss is more likely to be *permanent* if several weeks or months elapse after the injury before it becomes felt. Again, whether vision be temporarily or permanently impaired, directly or indirectly, immediately or remotely in time, depends upon the nature and extent of the injury and the pathological sequences which it induces.

The forms of visual disturbances may vary greatly according to the parts injured. If the muscles of the ball, or their nerve-supply, are the suffering parts, the normal and coördinating movements of the ball are prevented. There is then an inability to fix both eyes simultaneously upon the same object, or, in other words, binocular vision is interfered with, and diplopia, or double vision, takes place. This diplopia is very annoying, and often distressing, by calling forth such nervous phenomena as vertigo, staggering, nausea, and vomiting. It often compels the patient to desist from all efforts at binocular vision, and to look with one eye alone. In such cases the acuteness of vision may, however, be perfectly normal in each eye.

In case the intra-ocular, or ciliary muscle, or its nerve-supply suffers from injury, the pupillary movements are weakened or destroyed, the pupil is abnormally dilated, and there is an inability to focus or accommodate the eye for near objects. This loss of accommodation often causes great alarm to the patient, and he fears imminent blindness. But such loss does not cause blindness. If this were true, every normal eye would become blind after forty-five years of age, as ordinary presbyopia, or old-age sight, is but the loss of this power. If, through injury, an emmetropic, or normally refractive eye, becomes thus affected, vision for distances of twenty feet or more will still be found to be normal, and proper convex glasses will enable such an eye to see distinctly at any shorter distance. The acuteness of vision, then, remains unimpaired, and only the ability to change the focus has been diminished. If, however, an eye of a person under forty was hypermetropic or astigmatic before his accommodation had thus been destroyed, then the unaided vision becomes impaired for all distances, while before the injury it may have been fairly good, as the existing refractive error could be completely or in great measure overcome. It may be shown that the impaired vision is due to the refractive error, which loss of accommodation now prevents the patient from overcoming, by neutralizing the error with proper glasses. Then the vision becomes as acute, or perhaps more acute, than before the injury.

The existence of refractive errors in such cases is liable to lead the surgeon astray, unless they be carefully looked for and measured, and the vision tested under proper correction with glasses. The traumatically induced "asthenopia," or "weak vision," of the older observers was simply this loss of power of the ciliary muscle.

One eye alone being thus affected by an injury, the unequal focussing power of the two eyes renders binocular vision confusing and often very distressing.

These affections of the extra- and intra-ocular muscles of the eye do not, therefore, strictly impair the acuteness of vision, but often greatly disturb it, and, by reflex action, cause various nervous distresses.

Injuries of the eyeball itself may vary from total destruction by cuts, lacerations, and ruptures, to mere concussions, where no organic change can be detected, either externally or internally. The external parts may be wounded or contused in many ways, and at almost any point, and yet vision may be only temporarily impaired. Should the iris or vitreus prolapse through a wound, the case becomes complicated in view of secondary results, which might lead to irreparable loss of sight. The iris itself may be wounded with very little ill-results. Hemorrhages into the anterior chamber, and, indeed, into the vitreus, if not too large, will be absorbed,— the former very readily. Concussion or wound of the crystalline lens causes cataract, with "clouding" of vision, but this may, usually, be removed by some method of absorption or extraction, when, with the proper cataract glass, vision may be fairly good. Dislocation of the lens into the anterior chamber of the eye causes serious secondary effects, unless it is returned behind the iris or extracted through a corneal incision. Dislocation of the lens backwards into the vitreus produces the refractive condition of aphakia, and vision may be good at first with a cataract spectacle. But after months, or, perhaps, years, the lens acts as a foreign body in the bottom of the eye, and finally destroys vision. When the lens becomes extruded through a wound or rupture of the ball, so much damage is usually done to the parts as to cause entire loss of vision.

Injuries which destroy the transparency or normal curvature of the cornea, correspondingly and permanently impair sight. Traumatic iritis, or irido-choroiditis, with extensive exudation, seriously alters the mobility of the pupil, the transparency of the lens, and the nutrition of the eye, and thus causes permanent loss of sight. Excessive loss of vitreus or extensive hemorrhages into it, are very seldom fully recovered from. If the hemorrhages into the vitreus are small, however, they will be slowly absorbed, and after weeks or months the vision may be regained.

Ruptures, hemorrhages, or detachments of the retina or choroid cause blindness at the parts involved and beyond, along the course of the retinal fibers implicated. When the injury is at or near the macula lutea, sight suffers most. Injuries of these structures are seldom fully recovered from, and they often lead to total loss of vision in the eye affected. Sometimes a mere concussion of the retina, with no discernible structural change in the fundus, will impair vision very much, and even permanently.

An injury complicated by the lodgment of a foreign body within the eye, which cannot be extracted, generally leads to permanent blindness of the injured eye.

In most cases only one eye becomes injured. In case, however, that a foreign body is lodged within the eye and cannot be removed, or a lacerated or punctured wound involves the ciliary region, the loss of sight may not be confined to the injured eye, but the sound eye may become sympathetically diseased, and the vision of this, too, destroyed, the patient thus becoming totally blind.

Contusions or lacerations of the optic nerve from missiles or foreign bodies penetrating the orbit, dislocation of the ball from its socket, pressure on the nerve by fractured bone at the optic foramen, traumatic, orbital cellulitis, large hemorrhages into the optic-nerve sheath, all usually result in blindness of the corresponding eye. Other orbital injuries are generally without ill-results to sight.

Injuries may sometimes be inflicted, and for the most part, indirectly, upon the visual apparatus within the cranial cavity, and are, except in rare instances of large hemorrhages or extensive fractures at the base of the brain, limited to one side. An injury of the fibers constituting the visual path behind the optic commissure, whether in the optic tract, the basal ganglia, the internal capsule, the so-called " optic radiations," or the visual centers in the cortex of the occipital lobe, causes a form of blindness known as hemianopsia, or half-blindness, whose completeness is proportionate to the number of visual fibers injured or pressed upon. When all the fibers are involved, the patient is totally blind in just one-half of each field of vision, and in that half opposite to the path injured. This is due to the peculiar anatomical distribution of these fibers to the eyes, those from the left optic tract dividing at the optic commissure and going to the left half of each retina, and those of the right tract, to the right half of each retina. When this so-called lateral, homonymous hemianopsia exists after an injury, or from any other cause, the proof is almost positive that the lesion is in the course of the visual fibers, somewhere posterior to the optic commissure, or at the visual center in the occipital lobe, but its exact location must be determined by other attending symptoms. If that portion known as the optic tract is affected, the hemianopic pupil-reflex may be elicited. But if behind the basal ganglia, the pupil-reflex is normal. When this form of hemianopsia is present, the patient often declares that he is blind in one eye, but examination shows that he is "half-blind" in both eyes, and on the side of the supposed blind eye. The direct vision is usually fair, and the patient may be able to read the finest print, but continued close use of the eyes is attended by ocular fatigue. In walking or riding, he needs to turn his head frequently toward the affected side to avoid colliding with objects which he might not otherwise see. The ophthalmoscope reveals no change from the normal appearance in the fundus of either eye.

Injuries at the base of the brain, which do not at first affect the sight, may, later, by extension of meningeal inflammation, induce an optic neuritis, with subsequent atrophy, which may seriously impair it. The ophthalmoscope then depicts, at first, the optic disc reddened, with its outlines dimmed, and its circumference fringed with radiating lilac-colored or reddish streaks, and the retinal arteries narrowed and the veins distended and tortuous. In severe cases more marked appearances may be seen. After weeks or months the ophthalmoscopic picture is changed to that of optic atrophy, with the whitened disc with sharp outlines, and all the retinal vessels diminished in size.

Lastly, the vision, in rare instances, becomes affected by indirect influences, when no part of the visual apparatus has been injured. This most frequently occurs after injury to the spine, especially in the upper part of its extent. The ophthalmoscope may then show the optic nerve to be red and congested, or to be actively inflamed, exhibiting the picture of a mild neuritis. In the process of a long period of time, atrophy of the nerve may also take place, or, on the other hand, resolution may restore the parts to their normal condition. When blindness does occur from this cause, it is generally permanent, although exceptions to this have been reported. The vision is not always affected when the optic disc shows marked pathological changes.

Other ocular changes, besides those found in the optic nerve, are sometimes seen after spinal-cord injuries. These are found in the pupil in one or both eyes. In one patient it may be contracted, and in another, more or less dilated. The reflex of the pupil to light is usually lost in either case. In proportionately few instances, this class of spinal injuries, with or without optic nerve changes, leads to paralysis or paresis of one or more of the extra-ocular muscles.

It is supposed, and undoubtedly with truth, that the pupil-changes arise from the irritability or paralysis of the vaso-motor nerves, which come to the eye through the cervical sympathetic, the subordinate vaso-motor centers in the gray matter of the cord, and the vaso-motor fibers in the lateral columns of the cord, these having their origin in the chief, dominating, or general vaso-motor center situated in the medulla oblongata in the floor of the fourth ventricle.

The ophthalmoplegiæ of all kinds are due to disease of the nuclei of the nerves which supply the muscles of the ball, superinduced by vaso-motor influences initiated by spinal-cord injuries.

There seems to be some specific connection between the optic-nerve disease and spinal-cord disease. But as to how or where this connection exists, is still an undecided question. E. Berger, of Paris, (*Archives of Ophthalmology*, vol. xix., 1890, page 429), has recently published an extended review of the subject, and submits the results of his personal investigations. He believes that the optic-nerve lesion, and also the lesions in the posterior columns of the cord, are primarily due to vaso-motor disturbances at the vaso-motor centers for these structures, situated in the general vaso-motor center of the medulla, and that disease of these centers is caused by inflammation of the ependyma, or lining membrane, of the spinal cord and ventricles of the brain. He supports his position with a plausibility that at least commands consideration. His theory certainly explains many phenomena connected with ocular and other symptoms in spinal-cord degenerations from any cause, which could not, heretofore, be rationally accounted for.

Another indirect cause of blindness is supposed to be some injury of branches of the fifth, or trigeminal nerve, preferably its frontal branch. Many instances of this kind have been recorded, but, most singularly, these, for the most part, occurred in pre-ophthalmoscopic times. Such blindness has been explained by referring it to some indefinable reflex influence brought to bear upon the sympathetic nervous mechanism of the eye in such a manner as to alter seriously the blood circulation or the nutrition of the optic nerve and ball. I cannot deny the existence of such a cause; but in view of the fact that, with the ophthalmoscope, such cases do not appear, I am inclined to believe that in the old cases concussion of the retina, embolism of the retinal artery, hemorrhages or ruptures, could they have been seen, would have accounted for the existence of the "amaurosis."

This presentation, brief though it is, of the various injuries which may be inflicted upon the visual apparatus, and their most probable effects upon vision, shows, it seems to me, that the subject is of sufficient magnitude and importance to emphasize the necessity of giving careful and intelligent attention to this class of cases. In themselves, such injuries do not endanger the life of the individual, they do not furnish large pools of blood, and the tourniquet, Esmarch's bandage, or the amputating-knife and saw, do not figure in the treatment, but they often do rob living of one of the functions and powers most essential to its enjoyment and usefulness. It is, therefore, clearly the duty of the attendant not to look upon these cases lightly, not even those which appear trivial, as these often prove most serious.

A proper investigation and diagnosis of a case of injury to sight not only presuppose a fair knowledge of the anatomical, physiological, and pathological relation of the parts, but they require the use of certain instruments and methods, which should be in the possession of every general surgeon and physician. These should include, at least, a magnifying lens for focal illumination, an ophthalmoscope for examining the bottom of the eye, test-letters and test-objects for determining the acuteness of vision, and tests for outlining the field of vision. With these means at hand it should be the rule of the surgeon to determine the state of vision at the time of injury, if possible, and at frequent intervals afterwards for some time, as well as the condition of the ball itself as to the presence of wounds, hemorrhages, foreign bodies, etc. Attempts at malingering should be suspected and thwarted. Perhaps refractive errors must be corrected at the hands of an oculist before reliable information as to the exact acuteness of vision can be obtained. Moreover, the sight may have been poor before the accident, and hence a reliable previous history of the state of vision is exceedingly desirable, both from the patient and from second parties. For example, there are many cases of congenital amblyopia of one or both eyes, and particularly in cases of refractive error. The use of tobacco, alcohol, or large doses of quinine often produce visual disturbances of a pronounced character. Many chronic diseases of the fundus of the eyes cause loss of vision, and, if confined to one eye, may exist for a long time, and without the patient having become cognizant of it. All these conditions must be eliminated from the case in determining whether the injury is the true cause of blindness or not.

A correct diagnosis having been made, the course for the surgeon to pursue is first to prevent as little secondary damage as possible, and second, to administer to the subsequent pathological processes as they arise. When the ball is irretrievably lost, or a foreign body is lodged within it which cannot be extracted, he should enucleate or eviscerate it without delay. The responsibility of allowing a patient to go totally blind by sympathetic ophthalmia, through a vain effort to save an eye whose usefulness is totally destroyed, or by permitting the objections of unreasoning persons to prevail, is too great for any man to carry. The injury and blindness of one eye is a great calamity, but the calamity is far greater, and the blame almost inexcusable, when a lost eye is unnecessarily allowed to be the means of putting the patient in utter darkness the remainder of his life.

A large cut of the ball may be treated antiseptically, and the wound closed with fine silk sutures. Prolapsed iris should be excised at the first dressing, as it can scarcely ever be returned into the eye and kept there. Protruding vitreus may be excised and the wound closed by suture or compress. Wounds of the lens and iris should be treated with four- to eight-grain solutions of atropia. Sometimes the lens can be extracted through a linear incision in the cornea, when, after a few days, it has become generally opaque and softened. Foreign bodies impinging on the ball or optic nerve, or lodged within the ball or orbit, should, of course, be removed, if possible.

Very little can be done medically or surgically for internal injuries of the ball. Nature is often very kind and accomplishes much. In these cases we must rely mostly on her. Muscular palsies and optic-nerve affections are treated by mercurials, strychnia, electricity, and such other remedies as the nature of the case suggests.

212 Franklin Street.

